# Immunological Safety Evaluation of Exosomes Derived From Human Umbilical Cord Mesenchymal Stem Cells in Mice

**DOI:** 10.1155/sci/9986368

**Published:** 2025-04-26

**Authors:** Cancan Wang, Xinmei Hu, Yu Liu, Yu Xiao, Peng Jiang, Yunjing Lin, Xiaomin Liu, Zhengmian Zhang, Liang-cheng Li, Zhongquan Qi

**Affiliations:** ^1^Guangxi Key Laboratory of Special Biomedicine, School of Medicine, Guangxi University, Nanning 530004, China; ^2^Fujian Provincial Sperm Bank, Fujian Maternity and Child Health Hospital, Fuzhou 350001, China; ^3^School of Pharmaceutical Sciences, Xiamen University, Xiamen 361102, China

**Keywords:** effects, exosomes, general toxicity, hucMSC, immunotoxicity, safety evaluation, tail vein injection administration

## Abstract

Mounting evidence indicates that exosomes derived from human umbilical cord mesenchymal stem cells (hucMSCs-exosomes) combine the advantages of hucMSC pluripotency with their nanoscale dimensions, enhancing their clinical potential through prolonged circulation half-life. Despite these promising characteristics, research on their immunological toxicity remains insufficient. This study focuses on the impact of hucMSC-exosomes on the general toxicity and immunopathological indicators. When mice received tail vein injections of 6 × 10^10^ hucMSC-exosomes particles, we observed no significant changes in body weight, feed intake, blood composition, organ indices, or histopathological findings throughout the 14 days observation period. Similarly, blood levels of immunoglobulins, cytokines, and lymphocyte subpopulations remained stable. The hucMSC-exosomes produced no detectable negative effects on immune organs including the thymus, spleen, and bone marrow. These findings indicate that intravenous administration of 6 × 10^10^ particles of hucMSC-exosomes appears relatively safe at the murine level. This assessment of safety and immunological impact following intravenous hucMSC-exosomes infusion offers experimental support for potential clinical applications and future analyses in this field.

## 1. Introduction

Mesenchymal stem cells (MSCs) are recognized for their self-renewal abilities and differentiation potential [1]. Among the MSC family, human umbilical cord MSCs (hucMSCs) are highly valued for their multilineage differentiation capability, autologous transplantation feasibility, ease of acquisition, immune regulation, and ethical compliance [2–4]. However, limited numbers of transplanted hucMSC reach target tissues, exhibit low survival rates within hosts, and may cause capillary embolization and uncontrollable cell division when transplanted through veins [5, 6]. Therefore, an alternative approach that can match the reparative potential of hucMSC transplantation is necessitated.

Exosomes are small extracellular vesicles measuring about 30–150 nm in diameter and are secreted by diverse cell types through membrane fusion processes [7]. They carry biomolecules such as DNA, RNA, proteins, and lipids, reflecting their cellular origin [8]. Although early studies suggested that MSC injections could occasionally pose risks such as tumor growth, ossification, or calcification [9, 10], recent studies have shown that adverse events associated with MSC therapy are typically mild and transient, including fever, administration site reactions, constipation, fatigue, and sleeplessness, with no significant severe adverse events reported [11]. Exosomes effectively avoid challenges in conventional cell therapy approaches as a cell-free therapeutic option [12, 13] and hold promise as carriers for disease biomarkers such as Parkinson's disease and cancer [14]. Moreover, crucial protection for their molecular cargo against clearance mechanisms involving complement or macrophages can be offered due to their unique bilayer membrane configuration and microscopic dimensions, thereby extending circulation time and enhancing biological activity [7]. Additionally, exosomes exhibit low immunogenicity and have the ability to penetrate cellular membranes, making them attractive candidates for immunological applications, especially as they may target specific cell types [15]. Therefore, exosomes and the engineering of exosomes, which involves chemical or biological modifications of these lipid bilayer vesicles, hold promise for disease treatment.

Research has presented that hucMSC-exosomes possess several conserved characteristics, including low immunogenicity, remarkable self-renewal capacity, and immunoregulatory properties, without triggering tumorigenic outcomes [16, 17]. These exosomes demonstrate specific therapeutic potential across various conditions: they reduce creatinine and blood urea nitrogen levels in kidney diseases [18], promote macular hole healing in ocular disorders [19], and alleviate both acute liver injury and fibrosis [20]. In addition, studies indicate that hucMSC-exosomes stimulate Microglia to exert neuroprotective effects by enhancing anti-inflammatory cytokines such as TGF-*β* and IL-10, improving inflammation and increasing A*β* clearance [16]. In terms of immunology, hucMSC-exosomes regulate the immune system through multiple mechanisms. Research demonstrates their ability to modulate CD4^+^ T cell proliferation and early apoptosis, inhibit Th17 cell differentiation, and enhance Treg cell differentiation, finally restoring the Th17/Treg balance in patients suffering from Primary Sjögren's syndrome, a common autoimmune disease [21]. Besides, treatment with hucMSC-exosomes effectively mitigates graft-versus-host disease injury by reducing redox metabolism disorders and dampening inflammatory cytokine bursts in CD4^+^ T cells. Sun et al. [22] have conducted a comprehensive safety assessment of hucMSC-exosomes, supporting their potential as a promising therapeutic approach.

The immune system is integral to preserving the integrity of organisms. Beyond its primary function of safeguarding against pathogens, it is actively involved in the prevention, development, and defense against cancer [23]. The effect of exogenous substances on the immune system, which can manifest as either immunosuppression or immunoenhancement, may result in damage to normal cells and tissues, a phenomenon referred to as immunotoxicity [24]. In the past decades, extensive in vivo and in vitro studies have indicated the immunotoxic potential of various nanoparticles. While exosomes are classified as nanoparticles, the safety of hucMSC-exosomes in immunology has not been confirmed by articles. Drawing from this study, the present work directs its attention towards the effect of hucMSC-exosomes on immunotoxicity in animal models. The practical significance of this research is reflected by its contribution toward establishing an appropriate immune safety evaluation framework to finally advance clinical research in this field.

## 2. Materials and Methods

### 2.1. Statement of Ethics and Animal Treatment

Approval for the study protocol was granted by both the Medical Ethical Committee and the Animal Ethical Committee of Guangxi University (Approval No. GXU-2023-031 and GXU-2023-0234). All experimental procedures involving animals followed their guidelines for ethical conduct in animal care and use. We obtained 8-week-old C57BL/6 mice from SPF Biotechnology Co. Ltd. (Beijing, China) and housed them in a specific pathogen-free (SPF) facility environment. Throughout the study, mice were maintained under controlled conditions with a consistent 12-h light/dark cycle, temperature between 22 and 26°C, and humidity ranging from 50% to 70%. Animals had unlimited access to food and water for the entire duration of the experiment.

### 2.2. Culture of 3D-hucMSC and Characterization of hucMSC-Exosomes

For this study, hucMSC-exosomes were obtained from Professor Wang Yue's team at Naval Medical University. They were maintained at −80°C for long-term preservation or 4°C for short-term applications [25, 26].

A 3D hypoxia cell scale-up system with microcarriers and a 2.6 L bioreactor (BIRUI BIOTECHNOLOGY, China) was used for cell expansion culture. Passage 3 hucMSC were introduced into the bioreactor at a density of 8000–10,000 cells/cm^2^ and cultured under 5% hypoxia at 37°C with continuous medium supplementation. The culture supernatant was collected aseptically during and after cultivation, stored at 4°C for short-term and −80°C for long-term, for subsequent exosomes extraction.

To isolate exosomes from the culture supernatant, we employed a sequential combination of tangential flow filtration (TFF) and ultracentrifugation (UC). Initially, larger particles were removed through a series of centrifugation steps, followed by filtration through a 300 kD flat membrane. The processed supernatant then underwent two rounds of UC at 100,000 g for 70 min at 4°C. Finally, the exosomes suspension was concentrated, sterilized, and stored at −80°C for further experiments. A BCA protein quantitation kit (Zoman, Biotechnology, ZD301) was utilized to determine the protein content of the hucMSC-exosomes. Their morphology and size were analyzed utilizing an HT7700 transmission electron microscope (Hitachi, Japan). Nanoparticle tracking analysis (NTA; Malvern, UK) helped assess the particle size distribution. To identify marker proteins present in the hucMSC-exosomes, we conducted western blotting with antibodies against CD9 (Proteintech, 20597-1-AP), TSG101 (Proteintech, 28283-1-AP), HSP70 (abcam, ab2787) and the negative protein marker Calnexin (Proteintech, 10427-2-AP).

### 2.3. Hematology Assay

This study selected a cohort of 24 C57BL/6 mice, consisting of 12 males and 12 females, each initially weighing between 16 and 22 g. The mice were evenly divided into two groups, with an equal number of male and female subjects in each. The EXO group was subjected to tail vein injection of 6 × 10^10^ particles of hucMSC-exosomes, each diluted in 100 μL of PBS [20, 27–29]. Whereas the control group received injections of an equivalent volume of PBS. After 14 days postinjection, orbital blood samples were collected from the mice. For routine blood analysis, we extracted 100 μL of fresh blood into EDTA anticoagulation tubes from each mouse. An automatic blood cell analyzer (Mindray, China) was employed to measure several parameters: white blood cell (WBC) count, red blood cell (RBC) count, hematocrit (HCT), hemoglobin (HGB), platelet (PLT) count, PLT HCT (PCT), lymphocyte (Lymph) count, percentage of Lymphs (Lymph%), mean amount of RBC HGB (MCH), mean HGB concentration in RBCs (MCHC), mean RBC volume (MCV), mean PLT volume (MPV), neutrophil count (Gran), percentage of neutrophils (Gran%), monocyte (Mon) count, percentage of Mons (Mon%), RBC volume distribution width (RDW), and PLT volume distribution width (PDW). In addition, changes in T-cell and B-cell subsets, immunoglobulins, and cytokines in the blood were evaluated using an Acoustic Focusing Flow Cytometer (Invitrogen, USA) and a microplate reader (BioTek, USA). The flow cytometry analysis employed antibodies anti-CD4 (Invitrogen, 17-0042-82), anti-CD8 (Invitrogen, 12-0081-82), and anti-CD19 (Invitrogen, 25-0193-82). ELISA kits for IgA (Elabscience, E-EL-M0690c), IgM (Elabscience, E-EL-M3036), IgG (Elabscience, E-EL-M0692c), IFN-*γ* (Jianglai, JL10967), and IL-10 (Jianglai, JL20242) were adopted to detect the levels of immunoglobulins and cytokines.

### 2.4. Pathological Observation of hucMSC-Exosomes in Various Organs

Major organs were harvested and immediately fixed in 4% paraformaldehyde solution. After fixation, the organs were then rinsed with distilled water for 30 min, then sequential dehydration through a series of ethanol solutions (75%–100%). The tissues were then treated with xylene before embedding in molten paraffin to achieve transparency. Utilizing a microtome, these paraffin blocks were sectioned, smoothed with a heated iron, and mounted on glass slides. The preparations were then dried in an oven maintained at 68°C.

The tissue sections were subjected to deparaffinization with xylene, then, treated sequentially with ethanol (from high to low concentration) and finally with distilled water. Hematoxylin staining was proceeded for 10 min, succeeded by a rise in flowing water for 1 min, differentiation using a 0.25% hydrochloric acid alcohol solution for a few seconds, a further rinse with running water for 1 h, and a 10 min dehydration process. The sections were subsequently stained with an eosin alcohol solution in a graded ethanol series (from low to high concentration) for 3 min. Following dehydration with anhydrous alcohol, the sections were treated with xylene, covered with a glass cover slip, and sealed with rubber.

### 2.5. TUNEL Staining

For detection of apoptotic cells, the slide-mounted sections were first deparaffinized in xylene, and then, treated with a series of graded ethanol/water solutions. The sections were then subjected to TUNEL staining using a kit provided by Roche (11684817910). Following staining, the sections were coverslipped and visualized under confocal microscopy. Images captured from these preparations were analyzed by calculating the percentage of TUNEL-positive nuclei relative to total nuclei count, followed by statistical analysis of these data.

### 2.6. Immunofluorescence Analysis of CD4 and CD8

For detection of T-cell and B-cell subsets in spleen and thymus tissue, we employed immunofluorescence staining. Tissue sections were first cleaned through sequential immersion in xylene, absolute ethanol, and distilled water. Antigen retrieval consisted of heating sections in citric acid solution for 2 min, followed by natural cooling and triple washing with PBS (5 min per wash) on a decolorization shaker. Following a 30-min blocking period with 3% BSA, sections were incubated overnight with primary antibodies against CD4 (Invitrogen, 14-9766-82, 1:200) and CD8 (Invitrogen, 14-0808-82, 1:200) diluted in blocking buffer. Visualization was achieved utilizing Alexa Fluor-conjugated secondary antibodies (Invitrogen, A-11007, 1:500), while nuclei were highlighted with DAPI counterstain (Solarbio, C0065). Imaging was performed using a confocal microscope (Leica TCS-SP2-AOBS) and the observations were captured photographically.

### 2.7. Lymph Proliferation Assay

For the single-cell suspension from sterile spleen, the cells were prepared in RPMI 1640 complete medium. These cells were plated into 24-well flat-bottom plates at a density of 2 × 10^6^ cells/mL per well. Each experimental well received either 75 μL of Concanavalin A (ConA) solution (C2010, Sigma, 100 μg/mL) or lipopolysaccharide (LPS) solution (L8880, Solarbio, 400 μg/mL), while control wells were treated with an equal volume of PBS. After incubating the plates for 68 h at 37°C in a humidified atmosphere containing 5% CO2, we added 100 μL of CCK-8 (UElandy) to each well and continued incubation for an additional 3 h. Applying a microplate reader (BioTek, USA), we measured absorbance (Abs1) at 450 nm. Blank controls consisted of wells containing 75 μL of PBS, from which baseline absorbance (Abs0) was determined. The relative proliferation rate was then calculated according to the offered formula:

  $$\begin{array}{c} \text{Relative proliferation rate (\%)}=\text{ABs1}÷\text{ABs0}\times 100\%. \end{array}$$

### 2.8. Colony-Forming Unit (CFU) Assay

Following euthanasia, bone marrow was harvested from four femurs of normal mice by flushing with Iscove's MDM containing 2% FBS (StemCell Technologies, Vancouver, BC, Canada). The harvested material was strained through a 0.40 mm cell strainer (BD Falcon, Franklin Lakes, NJ, USA), followed by erythrocyte elimination utilizing red cell lysis buffer (R1010, Solarbio). We prepared an IMDM suspension containing 2 × 10^5^ nucleated cells/mL, with particles at concentrations of 10^7^, 10^8^, or 10^9^. Triplicate samples were then seeded into 35 mm Petri dishes (with an accompanying water dish) containing Methylcellulose-based medium supplemented with recombinant cytokines (including EPO) specific for mouse cells (MethoCult GF M3434, StemCell Technologies, Vancouver, BC, Canada). Following 11 days of incubation, we analyzed the cultures for various colony types: E (burst-forming unit erythroid [BFU-E]), GM (CFU-granulocyte-macrophage [CFU-GM], CFU-granulocyte [CFU-G], CFU-macrophage[CFU-M]), and mix (CFU-granulocyte, erythrocyte, megakaryocyte, and macrophage [CFU-GEMMz]). GM colony counts from control groups acted as blank controls (GM_S_0), while those from EXO groups were designated as experimental samples (GM_S_1). The GM inhibitory rate was calculated utilizing the following formula:

  $$\begin{array}{c} \text{Inhibitory rate (\%)}=\left(\text{GMs0}-\text{GMs1}\right)÷\text{GMs0}\times 100\%. \end{array}$$

### 2.9. Reye–Giemsa Staining

To prepare bone marrow smears, we aspirated mouse femurs utilizing a 1 mL syringe. Two drops of Reye–Giemsa A solution (Baso, Zhuhai, China) were applied to ensure uniform coverage of the specimen for 1 min. Then, four drops of Reye–Giemsa B solution were added and the solutions were gently mixed by creating ripples on the liquid surface with an ear washing ball. After an additional 2 min of staining, the smear was rinsed under running water and air-dried. Images were captured utilizing an inverted microscope (Olympus, CX31).

### 2.10. Statistical Analysis

Statistical analysis was performed utilizing GraphPad Prism 9 software for Mac. We evaluated group differences utilizing *T*-tests for two-group comparisons and one-way ANOVA when analyzing more than two groups. Results are expressed as mean ± SEM, with statistical significance defined as *p* < 0.05.

## 3. Results

### 3.1. Characterization of Exosomes

**Figure 1:**
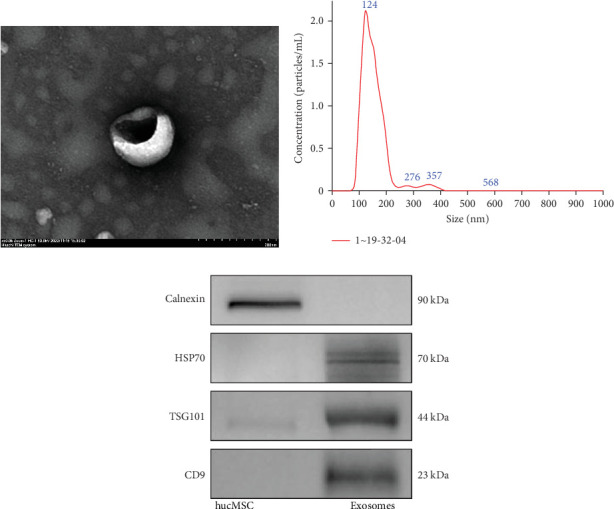
Characterization of exosomes derived from hucMSC. (A) Representative images of hucMSC-exosomes under a transmission electron microscope transmission electron microscopy (TEM). Bar = 200 nm. (B) Size distributions of hucMSC-exosomes were generated by nanoparticle tracking analysis (NTA). (C) Western blotting analysis of specific protein markers (CD9, TSG101, and HSP70) and negative protein marker (Calnexin) in hucMSC-exosomes.

Transmission electron microscopy (TEM), NTA, and western blotting were employed to characterize husMSCs-exosomes. Under TEM, hucMSC-derived exosomes exhibited a characteristic cup-shaped morphology (Figure 1A). Analysis by NTA indicated a particle population with a mode diameter of 123.7 nm at a concentration of 4.25 × 10^11^ particles/mL (Figure 1B). Western blotting indicated the presence of typical protein markers CD9, TSG101, and HSP70, but not Calnexin (Figure 1C). These findings confirmed the characteristics of hucMSC-exosomes and used in subsequent cellular and animal experiments.

### 3.2. Effect of hucMSC-Exosomes on Mouse Body Weight and Organ Coefficients

**Figure 2:**
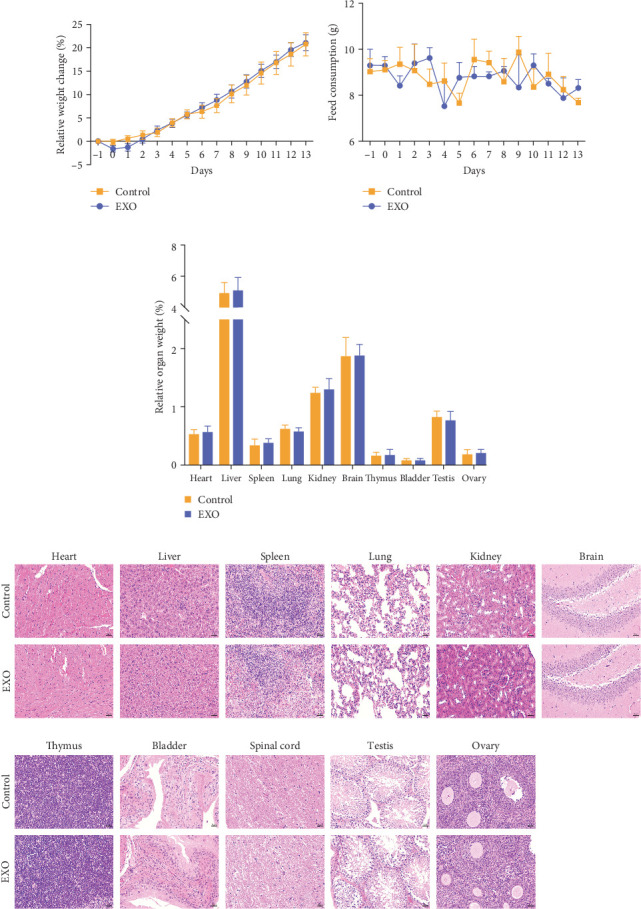
General toxicity indexes and pathological changes in mouse organs. (A) Body weight (*p* > 0.05), *N* = 12 per group. (B) Feed consumption (*p* > 0.05), *N* = 4 per group. (C) Relative organ weight (*p* > 0.05), *N* = 12 per group. Data are expressed the mean ± SEM. (D) HE-stained pathological sections of major organs in the control and hucMSC-exosomes group. *N* = 6 per group, bar = 50 μm.

To evaluate the effect of hucMSC-exosomes on physiological parameters, we monitored mouse body weight daily and tracked feed consumption per cage. Neither daily weight measurements nor feed intake demonstrated significant differences between the exosomes-treated and control groups (Figure 2A,B), with both groups displaying similar growth patterns. Following the 14-day experimental period, mice were euthanized for comprehensive organ analysis. We measured weights of the heart, liver, spleen, lungs, kidneys, brain, thymus, bladder, spinal cord, and reproductive organs (testicular or ovary), calculating relative organ weights for each. Compared to the normal group, mice receiving exosomes demonstrated no statistically significant differences in relative organ weights, including immune and reproductive organs (Figure 2C). Moreover, HE-stained pathological sections of the mouse organs were prepared to determine the presence of any influencing factors due to the injections. Finally, visual observation and pathological analysis demonstrated no pathological abnormalities or inflammatory cell infiltrates in the organ sections (Figure 2D).

### 3.3. Safety Evaluation of hucMSC-Exosomes in Terms of Hemocyte Parameters

**Figure 3:**
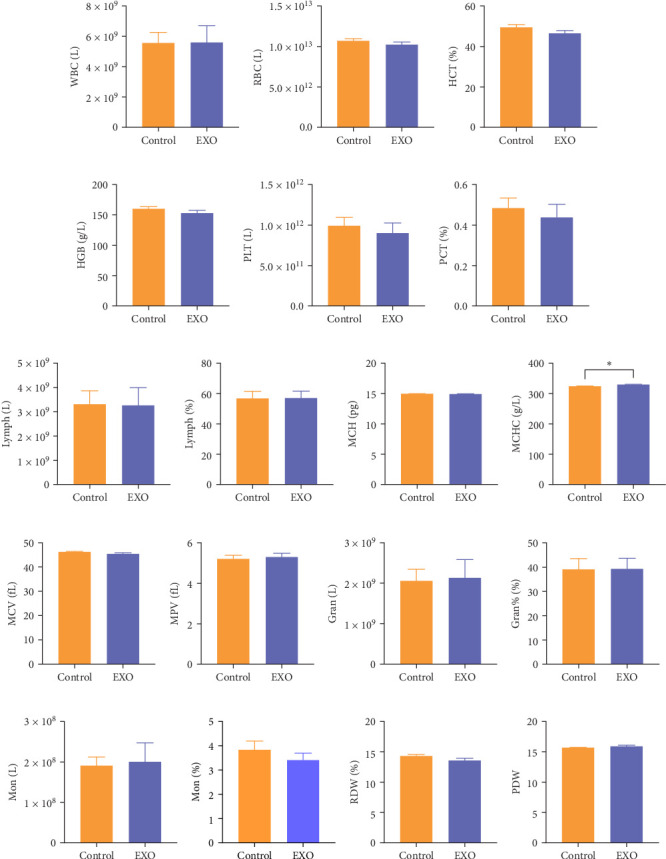
Routine blood analyses of mice. (A) White blood cell count. (B) Red blood cell count. (C) Hematocrit. (D) Hemoglobin. (E) Platelet count. (F) Platelet hematocrit. (G) Lymphocyte count. (H) Percentage of lymphocytes. (I) Mean amount of red blood cell hemoglobin. (J) Mean hemoglobin concentration of red blood cells (*⁣*^*∗*^*p*  < 0.05). (K) Mean red blood cell volume. (L) Mean platelet volume. (M) Neutrophil count. (N) Percentage of neutrophils. (O) Monocyte count. (P) Percentage of monocytes. (Q) Red blood cell volume distribution width. (R) Platelet volume distribution. *N* = 12 per group. Data are expressed the mean ± SEM.

To verify the effects of hucMSC-exosomes on blood composition, we injected mice with either hucMSC-exosomes or PBS (control group) through the tail vein. After a period of 14 days postadministration, we collected orbital blood samples for hematology analysis. Results demonstrated a statistically significant difference in MCHC between the control and EXO groups, whereas MCH and MCV remained similar between groups and no significant differences in HCT or RDW values were observed. While slight differences appeared in WBC, RBC, Gran, HGB, PLT, Mon, and Lymph counts between groups, these differences lacked statistical significance. Similarly, Lymph% and Gran% proportions remained consistent across groups. Parameters including PLT, MPV, PDW, and PCT also demonstrated no meaningful differences. Importantly, all blood parameter changes remained in normal reference ranges for healthy mice (Figure 3). These findings suggest that hucMSC-exosomes administered through tail vein injection do not adversely affect blood cells.

### 3.4. Effects of hucMSC-Exosomes on Apoptosis of Thymus and Spleen Cells

**Figure 4:**
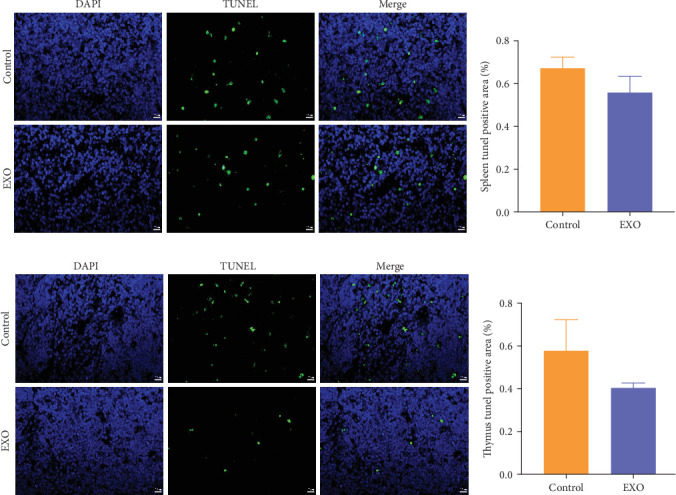
Effects of hucMSC-exosomes on apoptosis of thymus and spleen cells. (A) Representative picture of spleen TUNEL. (B) Quantitative detection of TUNEL-positive apoptotic area of spleen *N* = 6 per group (*p* > 0.05). (C) Representative picture of thymus TUNEL. (D) Quantitative detection of TUNEL-positive apoptotic area of thymus. *N* = 6 per group (*p* > 0.05). Data are expressed the mean ± SEM. DNA (blue) and TUNEL (green). Bar = 20 μm.

To investigate whether hucMSC-exosomes had an impact on the apoptosis of immune organ cells, paraffin sections were prepared and TUNEL staining was performed on the thymus and spleen of mice. Green signal was used to indicate TUNEL apoptotic cells. Our findings revealed that compared to the control group, a decrease in the proportion of apoptotic cells was observed after pretreatment with hucMSC-exosomes, but the difference was not significant (Figure 4A–D). This indicates that hucMSC-exosomes do not induce apoptosis in thymic or splenic cells.

### 3.5. Safety Evaluation of hucMSC-Exosomes in Terms of Immunoglobulin, Cytokines, and Lymph Phenotypes in Mice Peripheral Blood

**Figure 5:**
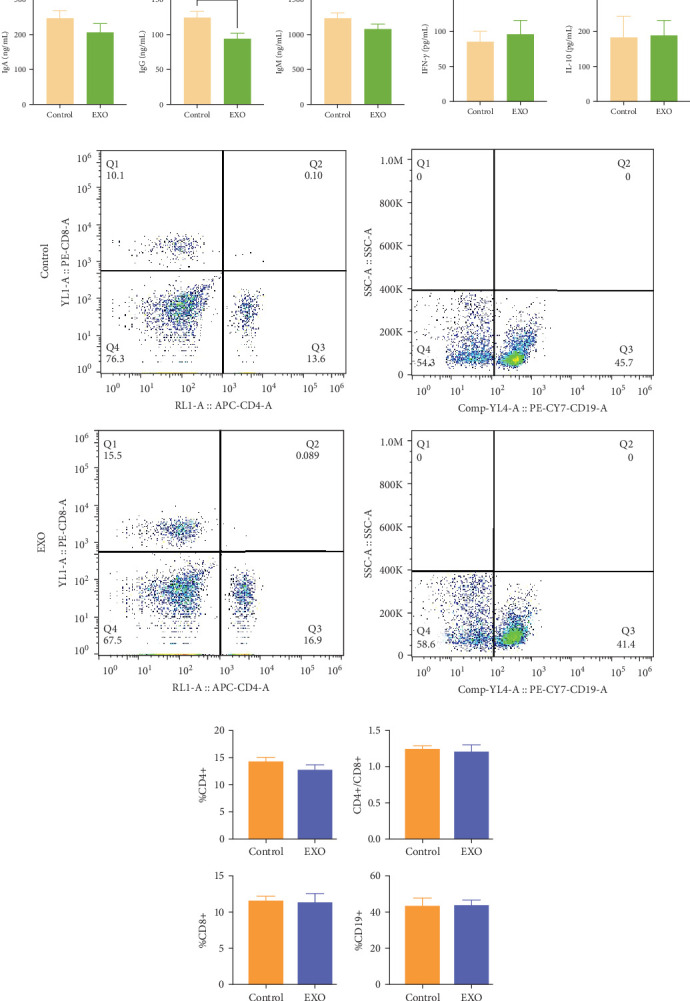
Effects of hucMSC-exosomes on immunoglobulin, cytokines, and lymphocyte phenotype in blood. (A) Changes of IgA, IgG (*⁣*^*∗*^*P* < 0.05), IgM, IFN-*γ*, and IL-10 contents in the two groups of mice by ELISA kit. *N* = 12 per group (*p* > 0.05). (B) Representative histograms of T-cell subsets and B-cell subsets of control and EXO group in flow cytometry. (C) Analysis of changes in the ratios of CD4^+^, CD8^+^, CD4^+^/CD8^+^, and CD19^+^. *N* = 12 per group (*p* > 0.05). Data are expressed as the mean ± SEM.

ELISA and flow cytometry were utilized to study effects on cytokine immunoglobulin, cytokines, and Lymph phenotypes in mice blood. A significant difference was observed in IgG content between the control and EXO groups. While the control group demonstrated slightly higher IgM and IgA content compared to the EXO group, this difference was not statistically significant. Similarly, no significant differences were determined in IFN-*γ* and IL-10 levels (Figure 5A). Flow cytometry measurements of T-cell and B-cell subset ratios (Figure 5B) indicated that CD4^+^ and CD8^+^ T-cells, CD4^+^/CD8^+^ ratio, and CD19^+^ B-cells remained similar between both groups (Figure 5C). These findings indicate that hucMSC-exosomes had no significant and negative effects on immunoglobulin, cytokines, or Lymph subsets in mice blood.

### 3.6. Safety Evaluation of hucMSC-Exosomes in Terms of Immune Organs of Spleen

**Figure 6:**
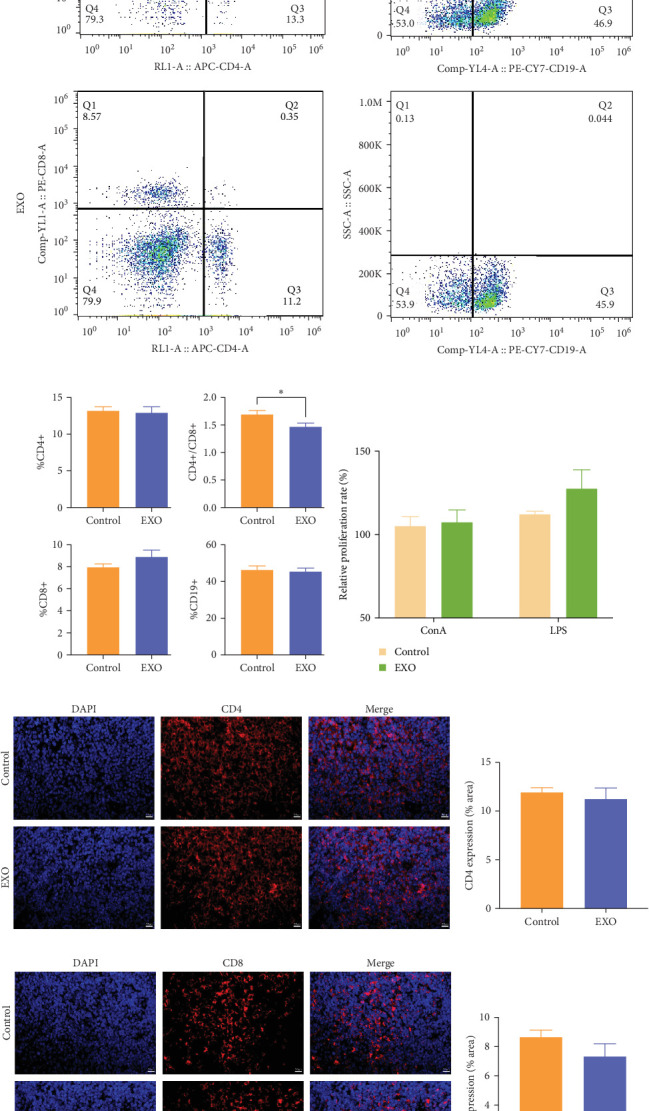
Effects of hucMSC-exosomes on lymphocyte phenotype and proliferation in spleen. (A) Representative histograms of T-cell subsets and B-cell subsets of control and EXO group in flow cytometry. (B) Analysis of changes in the ratios of CD4^+^, CD8^+^, CD4^+^/CD8^+^ (*⁣*^*∗*^*p*  < 0.05), and CD19^+^. *N* = 12 per group (*p* > 0.05). Data are expressed as the mean ± SEM. (C) ConA induced T cells and LPS induced B cells proliferation. Cell proliferation was determined using CCK8 assay. *N* = 12 per group (*p* > 0.05). Data are expressed as the mean ± SEM. (D) Representative histograms of immunofluorescence staining for CD4. (E) Analysis of changes in percentage of stained areas of CD4. *N* = 6 per group (*p* > 0.05). (F) Representative histograms of immunofluorescence staining for CD8. (G) Analysis of changes in percentage of stained areas of CD8. *N* = 6 per group (*p* > 0.05). Data are expressed as the mean ± SEM. Bar = 20 μm.

Flow cytometry was employed to appraise hucMSC-exosomes impact on lymphocyte subtypes in the spleen (Figure 6A). When comparing the experimental and control groups, CD4^+^ and CD8^+^ T-cell ratios and CD19^+^ B-cell ratios demonstrated no statistically significant differences. However, the CD4^+^/CD8^+^ ratio was statistically significantly lower in the EXO group than in control's (Figure 6B). Neither ConA-induced T Lymph proliferation nor LPS-induced B Lymph proliferation differed statistically significantly between groups (Figure 6C). Additional immunofluorescent staining for CD4 (Figure 6D) and CD8 (Figure 6F) distribution in mice spleens confirmed no statistically significant differences. These results suggest that hucMSC-exosomes did not adversely affect the spleen.

### 3.7. Safety Evaluation of hucMSC-Exosomes in Terms of Immune Organs of Thymus

**Figure 7:**
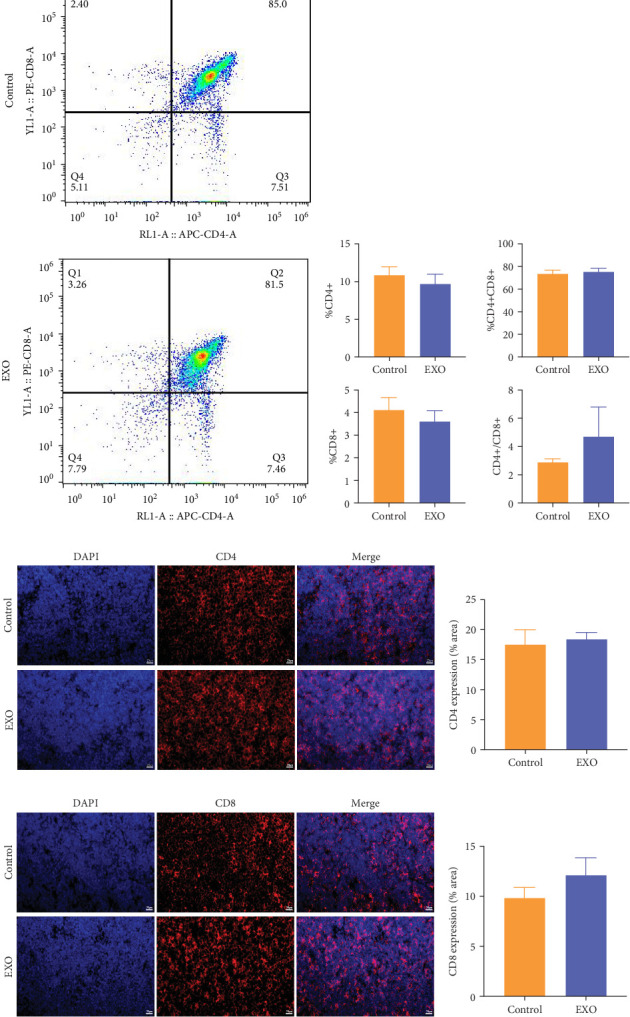
Effects of hucMSC-exosomes on T lymphocyte phenotype in thymus. (A) Representative histograms of T-cell subsets of control and EXO group in flow cytometry. (B) Analysis of changes in the ratios of CD4^+^, CD8^+^, CD4^+^CD8^+^ and CD4^+^/CD8^+^. *N* = 12 per group (*p* > 0.05). Data are expressed as the mean ± SEM. (C) Representative histograms of immunofluorescence staining for CD4. (D) Analysis of changes in mean fluorescence intensity of CD4. *N* = 6 per group (*p* > 0.05). (E) Representative histograms of immunofluorescence staining for CD8. (F) Analysis of changes in mean fluorescence intensity of CD8. *N* = 6 per group (*p* > 0.05). Data are expressed as the mean ± SEM. Bar = 20 μm.

For the thymus, flow cytometry analysis of T-cell and B-cell subset ratios (Figure 7A) demonstrated no statistically significant differences between experimental and control groups in CD4^+^, CD8^+^ T-cell ratios or the CD4^+^/CD8^+^ ratio. Immunofluorescent staining analysis of CD4 (Figure 7C) and CD8 (Figure 7E) expression in mice thymus aligned with these findings, indicating no statistically significant differences. This consistency between flow cytometry and immunofluorescence data supports the conclusion that hucMSC-exosomes produced no negative effects on the thymus.

### 3.8. Safety Evaluation of hucMSC-Exosomes in Terms of Immune Organs of Marrow

**Figure 8:**
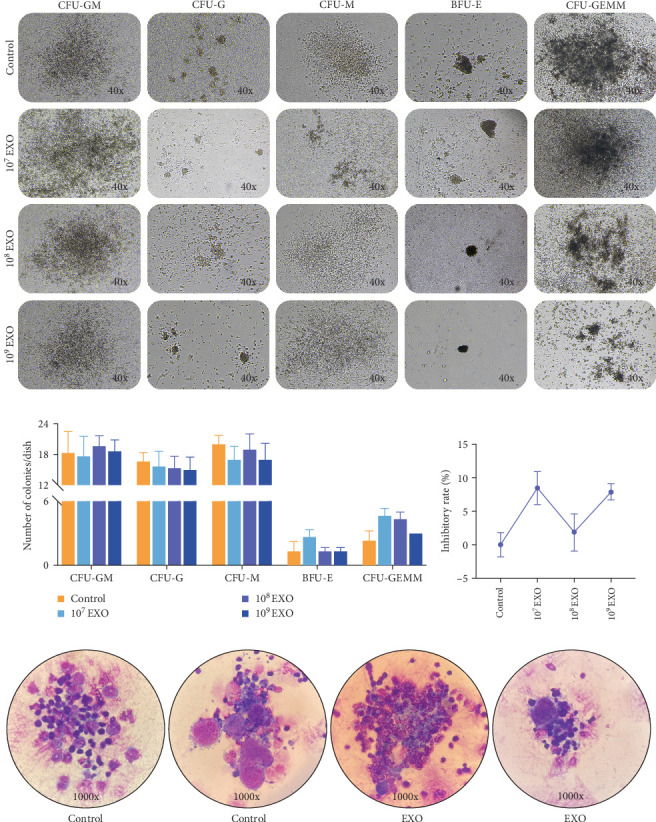
Effects of hucMSC-exosomes on bone marrow cells. (A) Representative photomicrographs of CFU-GM, CFU-G, CFU-M, BFU-E, and CFU-GEMM colonies after 11 days of 0, 10^7^, 10^8^, or 10^9^ particles of hucMSC-exosomes with mouse bone marrow cells cocultivation. Microscopic multiple with 40x. (B) Number of CFU-GM, CFU-G, CFU-M, BFU-E, and CFU-GEMM colonies in different groups. *N* = 3 per group (*p* > 0.05). (C) Analysis of the inhibitory rate of GM (CFU-GM, CFU-G, and CFU-M). *N* = 3 per group (*p* > 0.05). Data are expressed as the mean ± SEM. (D) Representative photomicrographs of bone marrow cell by Reye–Giemsa staining. Microscopic multiple with 1000x.

To analyze the potential impact of hucMSC-exosomes on bone marrow cell proliferation, CFUs were measured following coculturing with bone marrow cells for 11 days in vitro (Figure 8A). Between experimental and control groups, we determined no statistically significant differences in CFU-GM, CFU-G, CFU-M, BFU-E, CFU-GEMM, or GM inhibitory rates (Figure 8B). Utilizing Reye–Giemsa staining, we also analyzed both groups for morphology, volume, staining characteristics, and proportions of RBC lines, granulocyte lines, megakaryocytes, Mons, and Lymphs. All analyzed parameters appeared normal, with intact cell membranes and typical nuclear morphology (Figure 8C). These results suggest that hucMSC-exosomes neither negatively affected bone marrow nor produced dose-dependent effects.

## 4. Discussion

The therapeutic potential of exosomes in treating various clinical conditions has obtained significant attention in recent research. These vesicles represent the biophysical properties of MSCs while demonstrating greater effectiveness than the MSCs themselves [30]. Research suggests that hucMSC-exosomes exhibit particular promise for treating disorders affecting renal, hepatic, ocular, and neurological systems, including spinal cord injuries and neurodegenerative diseases [17]. The immune system often acts as the primary target for toxic substances, thus, triggering adverse reactions throughout the body. Variations in immunotoxicity offer particularly sensitive markers for evaluating the safety of exogenous materials. Our study conducted a systematic assessment of hucMSC-exosomes immunotoxic potential in mice. This evaluation comprised comprehensive analyses of general toxicity, blood immunopathology, and immune organ functionality. Our results indicated that even high-dose intravenous administration of hucMSC-exosomes produced no significant immunotoxicity in mice, lending support to their safety profile for further preclinical exploration.

First, to ensure experimental accuracy, we characterized hucMSC-exosomes, confirming their shape, size, and protein markers met exosomes definitions. Subsequently the tail vein of each mouse was injected with 6 × 10^10^ particles of hucMSC-exosomes. The injected amount separately was 9.4, 12, 4, 40, and 15 times that of the therapeutic dose reported in previous studies [20, 27–29]. During the observation period of 14 days after administration, the body weight changes and food intake of the mice were closely monitored and the results showed no significant differences compared with the control group, indicating that hucMSC-exosomes administration did not adversely affect the basic physiological status of the mice.

Then, we tested a series of general toxicity parameters, including organ weight indices, blood routine indicators, and histopathological sections. The results indicated all measurements fell in normal ranges with no significant changes. While the exosome group exhibited a slightly higher MCHC level, other hematological markers such as MCH and MCV remained similar between groups. All values stayed in established reference ranges for healthy mice, suggesting that hucMSC exosomes demonstrate minimal general toxicity in this animal model.

Since exosomes were administered through the tail vein, the circulatory system represented their primary point of contact. To evaluate potential immunotoxicity in blood, we performed ELISA tests for immunoglobulins and cytokines, while analyzing T-Lymph and B-Lymph subsets through flow cytometry. Figure 5 indicates significant differences in peripheral blood immunoglobulin IgG levels. This response likely occurs as hucMSC-exosomes, being of human origin, can be captured by murine immune cells, leading to an immune response and influencing immune status [12, 31]. Nevertheless, all other blood parameters remained unchanged between groups, aligning with previous safety and biodistribution studies of exosomes from human induced pluripotent stem cells [12]. These observations suggest hucMSC-exosomes likely pose no significant toxic effects on the murine blood immune system.

Finally, the study assessed the impact of hucMSC-exosomes on immune organs, namely, the spleen, thymus, and bone marrow, which constitute the immune system. Due to its high sensitivity to foreign substances, the immune system is considered to be the most vulnerable to toxic effects. To assess the effect of hucMSC-exosomes, several assays were performed, including tunel assay for apoptosis detection, flow cytometry for detecting T-Lymph and B-Lymph subsets, ConA-induced spleen T cell proliferation and LPS-induced spleen B cell proliferation, and immunofluorescence for CD4 and CD8 detection. Apoptosis is programmed cell death, which is crucial for controlling the number of cells in a multicellular organism [32]. Interestingly, we observed a trend toward decreased apoptotic cell proportions in both thymus and spleen tissues, though these reductions were not significant. This observation suggests hucMSC-exosomes may potentially offer protection against apoptosis. The proper functioning of the immune system depends heavily on maintaining balanced numbers and ratios of Lymph subsets [33]. Our findings indicated that hucMSC-exosomes administration did not disrupt this critical lymphoid subset homeostasis in either the spleen or thymus. In addition, when stimulated with either LPS or ConA, Lymph proliferation proceeded normally despite hucMSC-exosome exposure, aligning with observations from previous studies [34]. Additionally, the effect of hucMSC-exosomes on bone marrow cells was examined Reye's staining showed no significant abnormalities in the morphological proportions of various types of cells. The immunosuppressive experiment also demonstrated that hucMSC-exosomes did not inhibit bone marrow cell proliferation. Collectively, these findings suggest that hucMSC-exosomes are unlikely to induce immunotoxic effects in mice.

Although our study did not directly investigate mechanisms underlying the absence of immunotoxicity, prior literature suggests plausible pathways. One plausible explanation is that exosomes have the capability to augment the secretion of TGF-*β*, an anti-inflammatory factor, and facilitate the conversion of effector T cells into Tregs [35]. Besides, the presence of miR-146a-5 p and CD73 proteins in hucMSC-exosomes contributes to the downregulation of immunogenic signaling, further suppressing inflammatory and immune responses [36, 37]. This study has potential implications for the clinical utilization of hucMSC-exosomes. First, the safety study outcomes can inform the selection of hucMSC-exosomes dosages. By evaluating the safety of hucMSC-exosomes on a mouse model, the optimal range of dosages can be determined, thereby facilitating the establishment of an appropriate dosing regimen for clinical application. Furthermore, the assessment of immunogenicity and long-term safety of hucMSC-exosomes could be facilitated. Through evaluation of immune responses, researchers can better predict tolerance in human recipients and address safety concerns proactively. Finally, this research supports enhanced regulatory oversight of hucMSC-exosomes by informing the development of stringent safety evaluation criteria and regulatory frameworks, ensuring that any clinical use meets necessary safety standards.

Despite significant advancements in exosomes research, there remain numerous unresolved challenges that warrant further investigation. (1) The exploration of exosomes derived from diverse sources holds the potential to unveil novel functionalities and clinical applications. (2) The existing techniques for exosomes separation and purification exhibit certain limitations, including intricate procedures, suboptimal efficiency, and inconsistent yield. Enhancing these methods through optimization has resulted in improved yield and purity, thereby facilitating enhanced clinical applications. (3) Despite the preliminary findings obtained from mouse models, a comprehensive assessment of their safety and efficacy necessitates evaluation in larger preclinical models or clinical trials. (4) The advent of gene editing technology in recent years has presented novel prospects for the utilization of exosomes. Through the application of gene editing technology, it becomes feasible to modify the composition and functionality of exosomes, rendering them more tailored to specific clinical applications.

## 5. Conclusion

The results of this study indicated no significant deleterious effects from hucMSC-exosomes on general toxicity markers, including evaluations of physiological status, organ histopathology, and blood parameters. Similarly, when analyzing immunopathological indicators, we detected no toxicological changes. The treatment group receiving hucMSC-exosomes demonstrated similar levels to the control group across all measured parameters, including apoptosis, immune factors, Lymph subtypes, and immune-related protein expression. This study represents the first confirmation of safety for a single caudal vein administration of 6 × 10^10^ particles in C57BL/6 mice, with both general toxicity and immunotoxicity markers remaining favorable and no significant toxic reactions occurring. These findings offer empirical evidence supporting future exosome research and establish a foundation for potential clinical applications.

## Data Availability

The data that support the findings of this study are available on request from the corresponding author.
